# Sexual Dimorphism in the Pronotum and Elytra of *Dorcadion parilis* (Coleoptera: Cerambycidae): Evidence from Traditional and Geometric Morphometrics

**DOI:** 10.3390/insects17080760

**Published:** 2026-07-24

**Authors:** Aslı Doğan Sarıkaya

**Affiliations:** Department of Anthropology, Faculty of Arts and Sciences, Kırşehir Ahi Evran University, 40100 Kırşehir, Türkiye; aslidgn@gmail.com

**Keywords:** sexual dimorphism, geometric morphometrics, traditional morphometrics, morphological covariation, longhorn beetle

## Abstract

Sexual dimorphism may be expressed through absolute dimensions, relative proportions, and shape, yet these components have not been jointly evaluated in *Dorcadion parilis*, a Turkish endemic member of a flightless and taxonomically challenging genus. We examined 85 specimens (35 females and 50 males) using traditional and geometric morphometrics, sex-controlled allometric models, and two-block partial least squares analysis. Males had longer pronota, whereas females had wider pronota and longer and wider elytra. After Mosimann adjustment, the clearest differences were relatively longer pronota in males and broader elytra in females, while elytral length no longer differed. Pronotal and elytral shapes also differed between sexes, with stronger and more widespread differentiation in the pronotum and more localized elytral differences. Initial size–shape associations were not retained as independent effects after accounting for sex, and there was no evidence of sex-specific allometric slopes. Significant residual covariation between pronotal and elytral shapes persisted after adjustment for sex and structure-specific size. These findings show that sexual dimorphism in *D. parilis* is multidimensional and structure-specific rather than a simple consequence of centroid size and highlight the value of geometric morphometrics for detecting sex-related shape variation not fully captured by linear measurements and proportions.

## Simple Summary

In insects, sexual dimorphism may be expressed not only in the dimensions of body structures but also in their relative proportions and shapes. This distinction is especially important in *Dorcadion*, a flightless and taxonomically challenging genus in which pronotal and elytral characters are commonly used to distinguish species and compare populations. We examined 85 specimens of the Turkish endemic longhorn beetle *Dorcadion parilis*, focusing on the pronotum and elytra. Some differences in raw linear measurements weakened or disappeared after size adjustment, whereas relatively longer pronota in males and broader elytra in females remained the clearest traditional morphometric patterns. Geometric morphometrics revealed additional shape variation beyond these measurements and proportions. Pronotal shape showed stronger and more widespread differentiation between the sexes, whereas elytral differences were more localized. These shape differences remained evident after accounting for structure size, and pronotal and elytral shapes also varied in a coordinated manner. By distinguishing absolute dimensions, relative proportions, and shape, this study provides a more comprehensive account of sexual dimorphism in *Dorcadion parilis* and a basis for more reliable morphological interpretation in future taxonomic studies involving this species.

## 1. Introduction

Sexual dimorphism is a major component of intraspecific phenotypic variation and may be expressed through differences in the linear dimensions, relative proportions, and shape of anatomical structures. In insects, sexual size dimorphism has been widely documented and may vary within and among species in response to developmental and environmental conditions [1,2]. Beyond size-based differences, geometric morphometric studies in beetles have shown that differences between females and males may also be expressed through shape variation in external body structures [3,4,5]. Therefore, distinguishing absolute structural dimensions, relative proportions, and shape variation provides a more comprehensive framework for evaluating sexual dimorphism in morphologically diverse insect taxa.

Traditional morphometrics (TM) and geometric morphometrics (GM) provide complementary ways to examine these different components of sexual dimorphism. TM quantifies sexual dimorphism in linear dimensions and relative proportions. However, such variables cannot fully describe the spatial pattern or direction of shape variation [6,7]. GM captures this additional dimension by analyzing landmark configurations, allowing shape variation to be quantified and visualized as morphologically meaningful deformation patterns [6,7,8,9]. Because shape variation may covary with size, evaluating the relationship between shape and centroid size (CS) is also important before interpreting sexual shape dimorphism [5,10]. However, a size–shape relationship detected when females and males are analyzed together may reflect sex-related structuring of shape variation rather than an independent allometric effect. Therefore, models that explicitly evaluate size, sex, and their interaction provide a more robust framework for distinguishing allometric variation from sex-related shape differentiation.

The pronotum and elytra can reflect biologically meaningful variation in Coleoptera, including interspecific and intraspecific differences as well as sexual dimorphism in size and shape, as shown by TM and GM studies [4,11,12,13]. Comparative beetle morphometric studies further indicate that these structures may differ in the strength and pattern of their morphometric signals, with pronotal and elytral variation contributing differently to taxonomic, ecological, or sex-related differentiation [14,15,16,17,18]. Therefore, analyzing the pronotum and elytra separately is useful for determining whether sexual dimorphism is structure-specific or expressed similarly across dorsal body components. Beyond separate analyses, two-block PLS provides a framework for evaluating whether variation in pronotal and elytral shape is coordinated between structures rather than confined to structure-specific patterns [16,19,20]. In studies of sexual dimorphism, this framework can further be used to ask whether such covariation reflects shared sex-related variation or persists after accounting for sex-related effects [15,19].

Within Cerambycidae, the flightless genus *Dorcadion* provides a relevant context for examining sex-related variation in external morphology because it is well represented in the Turkish fauna [21,22] and belongs to a species-rich, taxonomically challenging lineage traditionally treated within Dorcadionini [23]. *Dorcadion parilis* Pesarini & Sabbadini, 2013 is a Turkish endemic species described from Oluközü, Yozgat Province, Central Anatolia [21,22,24]. The original description of *D. parilis* noted external morphological differences between sexes, particularly in elytral proportions and relative robustness [24]. Existing morphometric work on *Dorcadion* has mainly addressed taxonomic differentiation, variation among populations, or sexual dimorphism through either TM or GM analyses of selected structures. A traditional morphometric study of *D. axillare* used external measurements in a taxonomic context [25], whereas geometric morphometric studies of *D. anatolicum* and *D. micans* identified sexual dimorphism or morphological differentiation among populations based on pronotal size and shape [26,27,28]. Although the pronotum and elytra have been jointly analyzed in *D. micans*, that study assessed morphological differentiation among populations and taxa and included only male individuals to avoid variation associated with sexual dimorphism [29]. However, sexual dimorphism in *D. parilis* has not yet been evaluated through a combined approach including traditional morphometrics, geometric morphometrics, allometric assessment, and pronotum–elytra shape covariation.

To address this gap, the present study evaluates sexual dimorphism in the pronotum and elytra of *Dorcadion parilis* using traditional morphometrics and geometric morphometrics as complementary approaches. Specifically, we aimed to: (i) examine sex-related differences in pronotal and elytral linear dimensions and relative proportions using TM; (ii) evaluate sexual shape dimorphism and potential allometric effects in the pronotum and elytra using GM; and (iii) test pronotal–elytral shape covariation using two-block PLS.

## 2. Materials and Methods

### 2.1. Sampling and Species Identification

**Figure 1:**
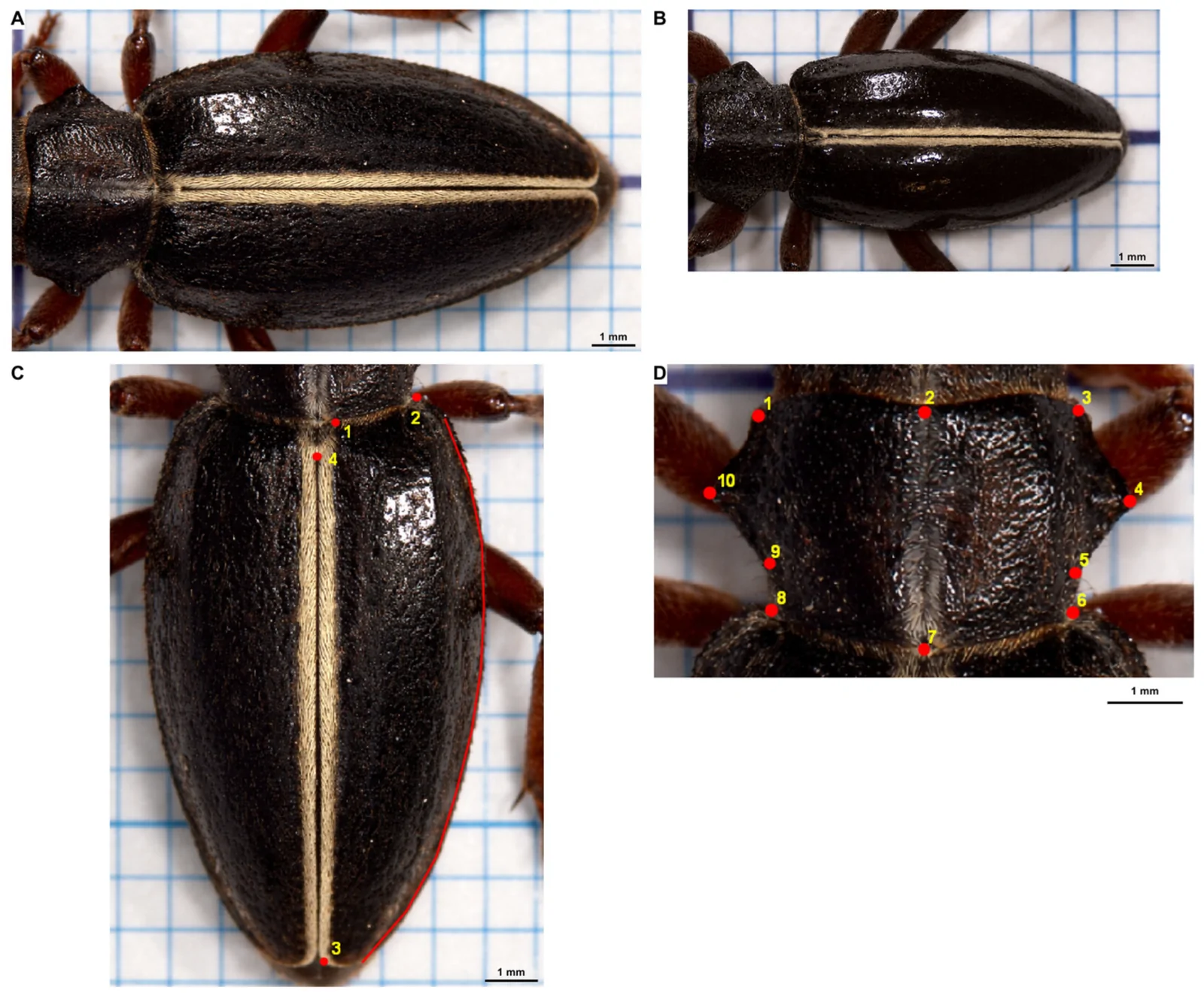
Representative dorsal views and landmark configurations of *Dorcadion parilis*. (**A**) Female and (**B**) male specimens shown at the same physical scale, with the pronotum and elytra clearly visible. (**C**) Right elytron showing four fixed landmarks (1–4); the red line indicates the digitized lateral contour used to generate six equally spaced curve-derived pseudolandmarks. (**D**) Pronotum configuration showing 10 fixed landmarks (1–10). Scale bars = 1 mm.

The specimens of *Dorcadion parilis* Pesarini & Sabbadini, 2013 were collected by hand during field surveys in open steppe habitats in the vicinity of Oluközü, Akdağmadeni District, Yozgat Province, Central Anatolia, Türkiye. The collection area is consistent with the known distribution of the species, which was originally described from the vicinity of Oluközü and has subsequently been reported from the same region [21,22,24]. Species identification was confirmed by H. Özdikmen based on the external diagnostic characters provided in the original species description. Sex was determined using fore-tarsal morphology and subsequently verified by gonadal inspection. The final dataset comprised 85 specimens, including 35 females and 50 males. All specimens were dry pinned, individually labeled, and deposited in the Entomology Collection of Kırşehir Ahi Evran University. Representative dorsal views of the pronotum and elytra in female and male specimens are shown in Figure 1A,B.

### 2.2. Specimen Imaging

All specimens were photographed in dorsal view using the integrated digital camera of a Leica EZ4 HD stereomicroscope (Leica Microsystems (Schweiz) AG, Heerbrugg, Switzerland). Specimens were positioned on millimeter-scale paper, which served as a scale reference for calibration. A single dorsal image was obtained for each specimen, and the same image was used for both linear measurements and geometric morphometric data acquisition. During imaging, specimens were oriented consistently along the longitudinal body axis to minimize variation caused by positioning. Images were checked for sharpness, scale visibility, and anatomical clarity before morphometric data collection.

### 2.3. Traditional Morphometric Data

Four linear measurements were obtained from dorsal images calibrated using millimeter-scale paper in Digimizer Image Analysis Software, version 6.4 (MedCalc Software Ltd., Ostend, Belgium): pronotal length (pL), maximum pronotal width (pW), elytral length (eL), and maximum elytral width (eW). All measurements were obtained in millimeters by a single observer to maintain consistency and avoid inter-observer variation.

Both raw measurements and Mosimann-adjusted proportional variables were used in the analysis. Mosimann-adjusted variables were calculated by dividing each linear measurement by the geometric mean of the four measurements for the same specimen [30], resulting in pL_adj, pW_adj, eL_adj, and eW_adj. Raw measurements describe absolute linear dimensions, whereas Mosimann-adjusted variables describe relative proportions.

### 2.4. Geometric Morphometric Data Acquisition

Geometric morphometric data were collected separately for pronotal and elytral shape configurations. Landmark and curve digitization was performed in tpsDig2, version 2.32 [31], and curve-derived pseudolandmarks were appended to the landmark configurations in tpsUtil, version 1.83 [32]. For the pronotum, 10 fixed landmarks were digitized to represent the anterior margin, anterolateral corners, lateral tubercles, posterolateral regions, and posterior median point. For elytral shape, the right elytron was used to standardize landmark digitization across specimens. Four fixed landmarks were placed on anatomically identifiable reference points corresponding to the basal, sutural, lateral, and apical regions of the right elytron. In addition, the lateral contour of the right elytron was digitized as a curve in tpsDig2 and represented by six equally spaced curve-derived pseudolandmarks. These pseudolandmarks were then appended to the fixed landmarks using the “append TPS curve to landmarks” function in tpsUtil, allowing them to be read as landmarks and resulting in a 10-point elytral configuration used in subsequent analyses.

All specimens were digitized using the same structure-specific landmark configurations and digitization protocol. The right elytron and pronotum landmark configurations used in the analyses are shown in Figure 1C and Figure 1D, respectively.

### 2.5. Measurement Error and Repeatability

To assess intra-observer repeatability of the linear measurements, a balanced subset of 20 specimens, including 10 females and 10 males, was measured twice by the same observer in two separate measurement sessions using the same dorsal images and anatomical measurement definitions. Each image was independently recalibrated in each session using the 1 mm reference provided by the millimeter-scale paper. The second measurement session was conducted independently, without reference to the values obtained in the first session. Repeatability was evaluated separately for pL, pW, eL, and eW using a two-way mixed-effects, single-measurement, absolute-agreement intraclass correlation coefficient [ICC(A,1)] with 95% confidence intervals. The technical error of measurement (TEM), relative technical error of measurement (rTEM), and mean between-session difference were also calculated for each trait.

Measurement error associated with landmark digitization was assessed separately for the pronotal and elytral configurations using Procrustes ANOVA in MorphoJ, version 1.08.02 [33]. For this purpose, a subset of 20 specimens, including 10 females and 10 males, was digitized twice by the same observer using the same structure-specific landmark configurations and digitization protocol. Procrustes ANOVA was used to partition variation into among-individual variation and digitizing error for both centroid size and shape. This procedure was used to assess the relative magnitude of digitizing error compared with among-individual variation.

### 2.6. Statistical Analysis

Traditional morphometric and geometric morphometric analyses were conducted to evaluate sex-related differences in pronotal and elytral morphology and to assess pronotum–elytra shape covariation. Traditional morphometric analyses, including the intra-observer repeatability assessment, were performed in R v4.5.2 (R Foundation for Statistical Computing, Vienna, Austria) using base R statistical functions [34]. Geometric morphometric analyses were conducted in MorphoJ and R, with R-based analyses performed using the geomorph v4.1.0 [35,36] and RRPP v2.1.2 packages [37,38]. Graphical outputs were prepared in R using ggplot2 v4.0.1 [39] and exported at high resolution.

Unless otherwise specified, permutation-based analyses in R were conducted using 9999 random permutations. DFA permutation tests conducted in MorphoJ were based on 1000 permutation runs, and significance values from MorphoJ permutation tests were reported as *p* < 0.001 when appropriate. To account for multiple testing in the follow-up univariate ANOVAs, *p*-values were adjusted using the Benjamini–Hochberg false discovery rate procedure. The adjustment was applied separately within each of four related sets of tests: raw linear variables, Mosimann-adjusted variables, pronotal PC scores, and elytral PC scores.

#### 2.6.1. Traditional Linear Morphometric Analyses

Raw measurements and the Mosimann-adjusted variables described above were analyzed separately. Multivariate analysis of variance (MANOVA), using Pillai’s trace as the test statistic, was used to test the overall effect of sex for each variable set. When significant multivariate sex effects were detected, follow-up univariate ANOVAs were examined for each trait to identify the variables contributing to sexual differentiation. Effect sizes for univariate tests were reported using eta squared (η^2^).

#### 2.6.2. Geometric Morphometric Analyses

The pronotal and elytral configurations described above were imported separately and subjected to Generalized Procrustes Analysis to remove non-shape variation associated with position, orientation, and scale. Procrustes shape coordinates and centroid size were obtained separately for each landmark configuration.

Allometric effects were assessed separately for pronotal and elytral shape using log-transformed centroid size (log(CS)) as the size variable. First, multivariate regression of Procrustes shape coordinates on log(CS) was used to evaluate the overall size–shape relationship. To determine whether this relationship reflected an independent effect of size rather than sex-related shape differentiation, sex-controlled Procrustes ANOVA models were then applied. Because sequential sums of squares were used, the additive terms were entered in alternative orders in separate models to test the effect of log(CS) after accounting for sex and the effect of sex after accounting for log(CS). The log(CS) × sex interaction was tested using the full factorial model. This approach allowed independent allometric effects to be distinguished from apparent size-related patterns arising from sexual shape dimorphism.

Principal Component Analysis (PCA) was performed on Procrustes shape coordinates separately for pronotal and elytral shape configurations to summarize the major axes of shape variation. To test sex-related shape differences, MANOVA was conducted using the principal components cumulatively explaining more than 90% of total shape variance. Accordingly, the first seven PCs were retained for pronotal shape and the first six PCs for elytral shape. Pillai’s trace was used as the multivariate test statistic. Follow-up univariate ANOVAs were then performed on individual PC scores to identify the components contributing most strongly to sex-related shape differentiation. As a complementary permutation-based assessment, residual randomization in a permutation procedure (RRPP) was used to test the effect of sex on the matrix of retained PC scores.

Discriminant Function Analysis (DFA) was used to evaluate sex-related shape separation for pronotal and elytral shape. Differences between female and male mean shapes were quantified using Procrustes and Mahalanobis distances, and their statistical significance was assessed using permutation tests. Hotelling’s T^2^ was also used as a parametric test of group separation. Classification accuracy was calculated using both the original discriminant function and cross-validation. Cross-validated discriminant function scores were used for DFA plots. Mean shape differences between females and males were visualized using wireframe comparisons of mean Procrustes configurations.

#### 2.6.3. Pronotal–Elytral Shape Covariation Analyses

Covariation between pronotal and elytral shape was evaluated using two-block partial least squares (PLS) analysis. Pronotal and elytral Procrustes shape coordinates were treated as two separate shape blocks and matched by specimen identity. An unadjusted PLS analysis was first used to evaluate overall covariation between the two shape blocks.

To evaluate whether pronotal–elytral shape covariation was influenced by sex-related shape variation, additional adjusted PLS analyses were conducted. First, a sex-adjusted PLS analysis was performed after residualizing each shape block against sex. Second, a sex- and size-adjusted PLS analysis was conducted by residualizing the pronotal block against sex and pronotal log(CS), and the elytral block against sex and elytral log(CS), before repeating the PLS analysis. In both adjusted analyses, the residual shape coordinates obtained from these block-specific models were used as the input blocks for the corresponding PLS analyses. For each PLS analysis, the PLS1 singular value, the percentage of total between-block squared covariance represented by PLS1, the correlation between pronotal and elytral PLS1 scores, and permutation-based significance values were reported.

## 3. Results

### 3.1. Traditional Linear Morphometrics

The linear measurements showed high intra-observer repeatability. Absolute-agreement ICC estimates were 0.886 for pL (95% CI: 0.710–0.955), 0.967 for pW (95% CI: 0.915–0.987), 0.942 for eL (95% CI: 0.835–0.978), and 0.987 for eW (95% CI: 0.960–0.995). The corresponding TEM values were 0.053, 0.070, 0.161, and 0.076 mm, and rTEM values were 2.41%, 1.95%, 2.12%, and 1.68%, respectively. Mean values in the second measurement session were lower by 0.034–0.108 mm across traits. Overall, measurement error was small relative to the magnitude of the recorded traits.

Linear morphometric analyses based on raw measurements and Mosimann-adjusted proportional variables revealed clear sex-related differences in pronotal and elytral dimensions. A multivariate analysis of variance (MANOVA) based on raw measurements showed a highly significant effect of sex on pronotal and elytral measurements (Pillai’s trace = 0.883, F (4, 80) = 150.74, *p* < 0.001). After Benjamini–Hochberg adjustment, all four follow-up univariate ANOVAs remained significant (all BH-adjusted *p*-values < 0.001). Univariate analyses showed that males had greater pronotal length (pL), whereas females exhibited higher values for pronotal width (pW), elytral length (eL), and elytral width (eW). Among these variables, elytral width showed the largest effect size (η^2^ = 0.67), indicating that this trait contributed most strongly to the observed raw-measurement differences (Table 1).

MANOVA performed on size-adjusted (Mosimann) variables (pL_adj, pW_adj, eL_adj, eW_adj) revealed a highly significant effect of sex (Pillai’s trace = 0.879, F (4, 80) = 145.95, *p* < 0.001), indicating substantial proportional differentiation between females and males. After Benjamini–Hochberg adjustment, pL_adj, pW_adj, and eW_adj remained significant (all BH-adjusted *p*-values < 0.001), whereas eL_adj remained non-significant (BH-adjusted *p* = 0.180). Effect sizes showed marked variation in the strength of sexual dimorphism across traits. Pronotal length (pL_adj; η^2^ = 0.81) and elytral width (eW_adj; η^2^ = 0.87) exhibited very large effects, demonstrating pronounced proportional differentiation. In contrast, elytral length (eL_adj) showed a negligible effect (η^2^ = 0.02), indicating that the raw difference in elytral length largely reflected the common size component rather than proportional differentiation.

**Figure 2:**
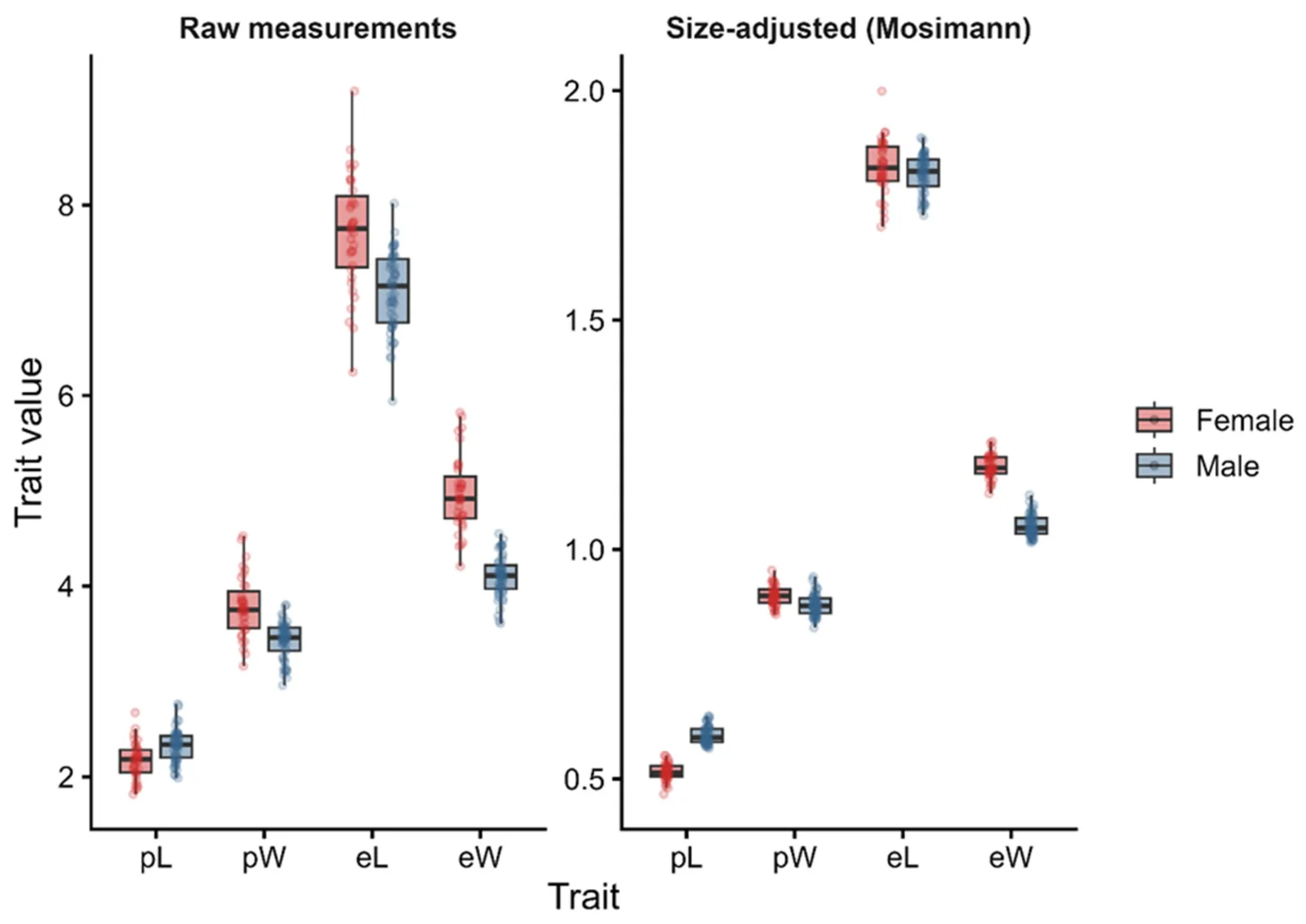
Distribution of raw linear measurements and Mosimann-adjusted proportional variables in female and male *Dorcadion parilis*. The left panel shows raw measurements, whereas the right panel shows size-adjusted proportional variables.

These patterns are summarized in Figure 2. Raw measurements showed sex-related differences across all four linear traits, with males exhibiting greater pronotal length and females showing greater pronotal width, elytral length, and elytral width. In contrast, Mosimann-adjusted variables showed a more trait-specific pattern: proportional differences were pronounced for pronotal length and elytral width, weaker for pronotal width, and not statistically significant for elytral length.

### 3.2. Geometric Morphometrics

To further assess sexual dimorphism in pronotal and elytral shape beyond linear measurements, geometric morphometric (GM) analyses were performed.

#### 3.2.1. Measurement Error

Measurement error associated with digitization was evaluated using Procrustes ANOVA for both pronotum and elytra. For centroid size, the effect of individuals was highly significant and substantially larger than digitizing error in both structures (pronotum: F = 465.13; elytra: F = 814.48; all *p* < 0.0001).

For shape, Procrustes ANOVA showed a strong individual effect for both pronotum (F = 24.05, *p* < 0.0001) and elytra (F = 21.60, *p* < 0.0001). In contrast, digitizing error represented only a small fraction of total shape variation, accounting for 3.8% in the pronotum and 4.6% in the elytra. These results indicate that inter-individual variation exceeded measurement error, confirming high repeatability of digitization and supporting the reliability of subsequent geometric morphometric analyses.

#### 3.2.2. Pronotal Shape Variation

A multivariate regression of pronotal Procrustes shape coordinates on log-transformed centroid size (log(CS)) initially indicated a significant size–shape relationship, with log(CS) explaining 13.84% of total pronotal shape variation (*p* < 0.0001). However, in the sex-controlled Procrustes ANOVA, log(CS) was no longer significant after accounting for sex (*p* = 0.6851), whereas sex remained highly significant after controlling for log(CS) (R^2^ = 0.4471, *p* = 0.0001; Table 2). The log(CS) × sex interaction was not significant (*p* = 0.8858), indicating no evidence for sex-specific allometric slopes. Thus, the apparent size–shape association was not supported as an independent allometric effect after accounting for sex.

**Figure 3:**
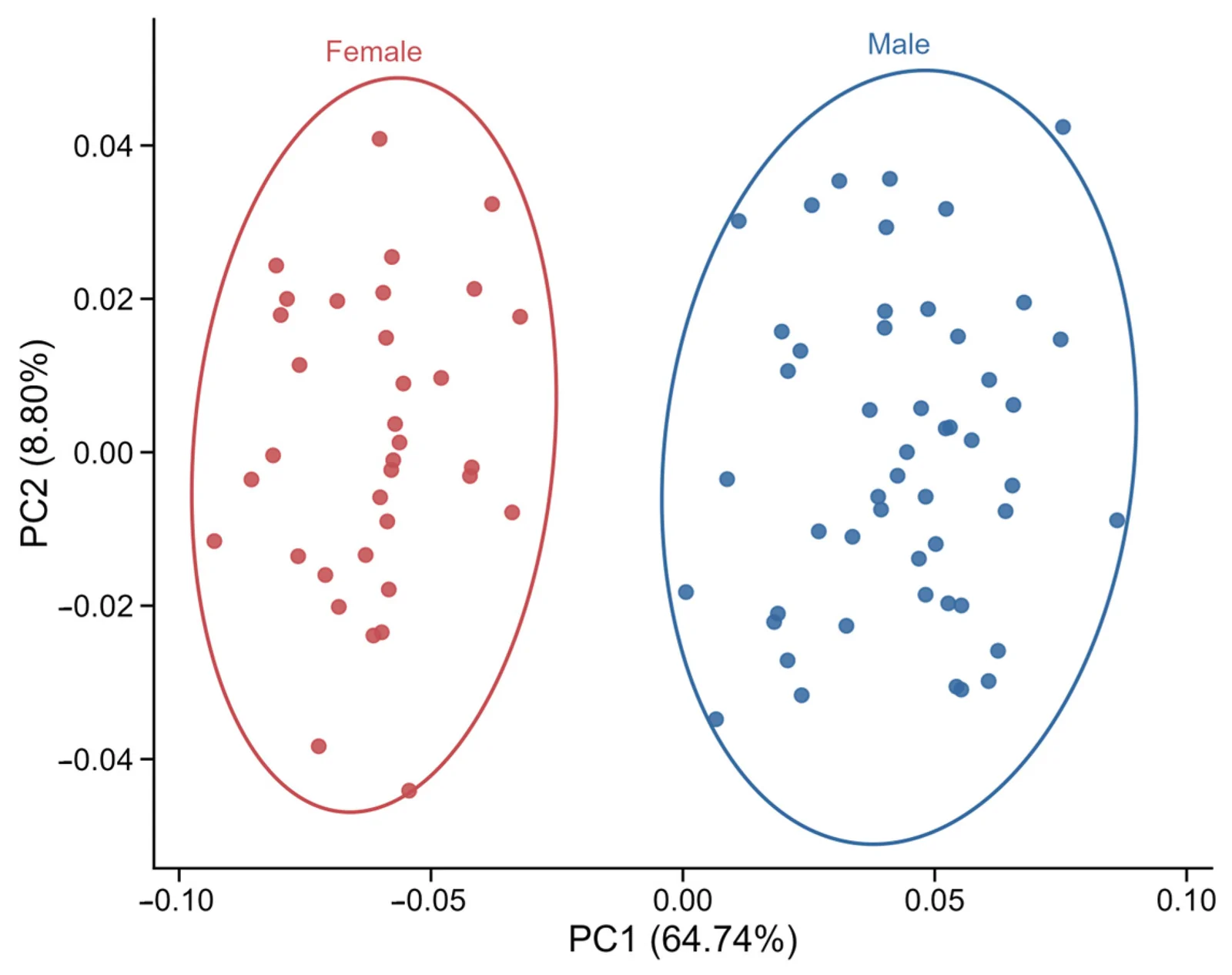
Principal Component Analysis (PCA) of pronotal Procrustes coordinates in female and male *Dorcadion parilis*. Ellipses represent 95% group-dispersion regions.

Principal Component Analysis (PCA) of pronotal Procrustes coordinates revealed that shape variation was strongly concentrated along the first principal component (Figure 3). PC1 explained 64.74% of total pronotal shape variance, whereas PC2 explained 8.80%, together accounting for 73.54% of the total variation. The first seven principal components cumulatively explained 90.14% of shape variance. The PCA scatter plot showed clear sex-related structuring of pronotal morphospace, with females and males mainly separated along PC1. In contrast, variation along PC2 did not show a distinct sex-related pattern.

Multivariate analysis of variance (MANOVA), performed on the first seven principal components explaining 90.14% of cumulative pronotum shape variance, confirmed a highly significant effect of sex on pronotal shape (Pillai’s trace = 0.914, F (7, 77) = 116.73, *p* < 0.0001). After Benjamini–Hochberg adjustment across PC1–PC7, the multivariate effect remained attributable primarily to PC1 (F = 726.09, BH-adjusted *p* <0.0001, η^2^ = 0.897), whereas PC2–PC7 remained non-significant after adjustment. A permutation-based RRPP analysis yielded a consistent result (F = 150.98, R^2^ = 0.645, *p* = 0.0001).

**Figure 4:**
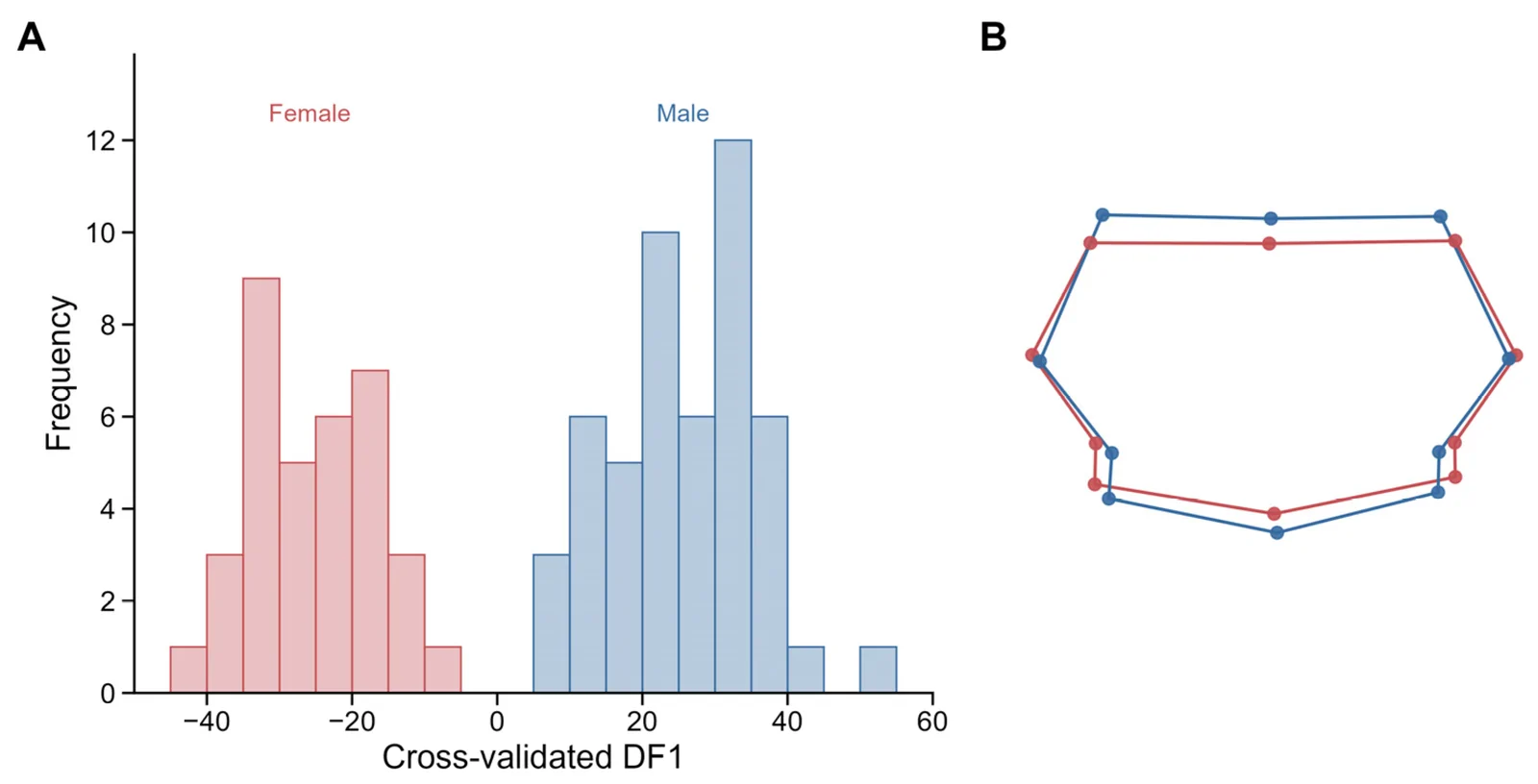
Discriminant Function Analysis (DFA) of pronotal shape in female and male *Dorcadion parilis*. (**A**) Distribution of cross-validated discriminant function scores. (**B**) Wireframe comparison of female and male mean Procrustes configurations. Red and blue indicate the female and male mean configurations, respectively.

DFA confirmed clear separation between female and male pronotum shapes. Mean shapes differed significantly based on Procrustes distance (0.1043, permutation *p* < 0.001) and Mahalanobis distance (7.2155, permutation *p* < 0.001), with a significant Hotelling’s T^2^ test (T^2^ = 1071.90, parametric *p* < 0.001). Cross-validated discriminant function scores showed complete separation between sexes (Figure 4A), and all individuals were correctly classified both in the original classification and under cross-validation.

Wireframe comparison of female and male mean Procrustes configurations (Figure 4B) showed that the male mean configuration had greater anterior–posterior extension, whereas the female mean configuration was more compact along this axis. Shape differences were most evident along the anterior margin and the posterolateral and posterior regions of the pronotum.

#### 3.2.3. Elytral Shape Variation

A multivariate regression of elytral Procrustes shape coordinates on log-transformed centroid size (log(CS)) initially indicated a significant size–shape relationship, with log(CS) explaining 14.99% of total elytral shape variation (*p* = 0.0001). However, in the sex-controlled Procrustes ANOVA, log(CS) was no longer significant after accounting for sex (*p* = 0.0657), whereas sex remained highly significant after controlling for log(CS) (R^2^ = 0.2236, *p* = 0.0001; Table 3). The log(CS) × sex interaction was not significant (*p* = 0.7566), indicating no evidence for sex-specific allometric slopes. Thus, the apparent size–shape association was not supported as an independent allometric effect after accounting for sex.

**Figure 5:**
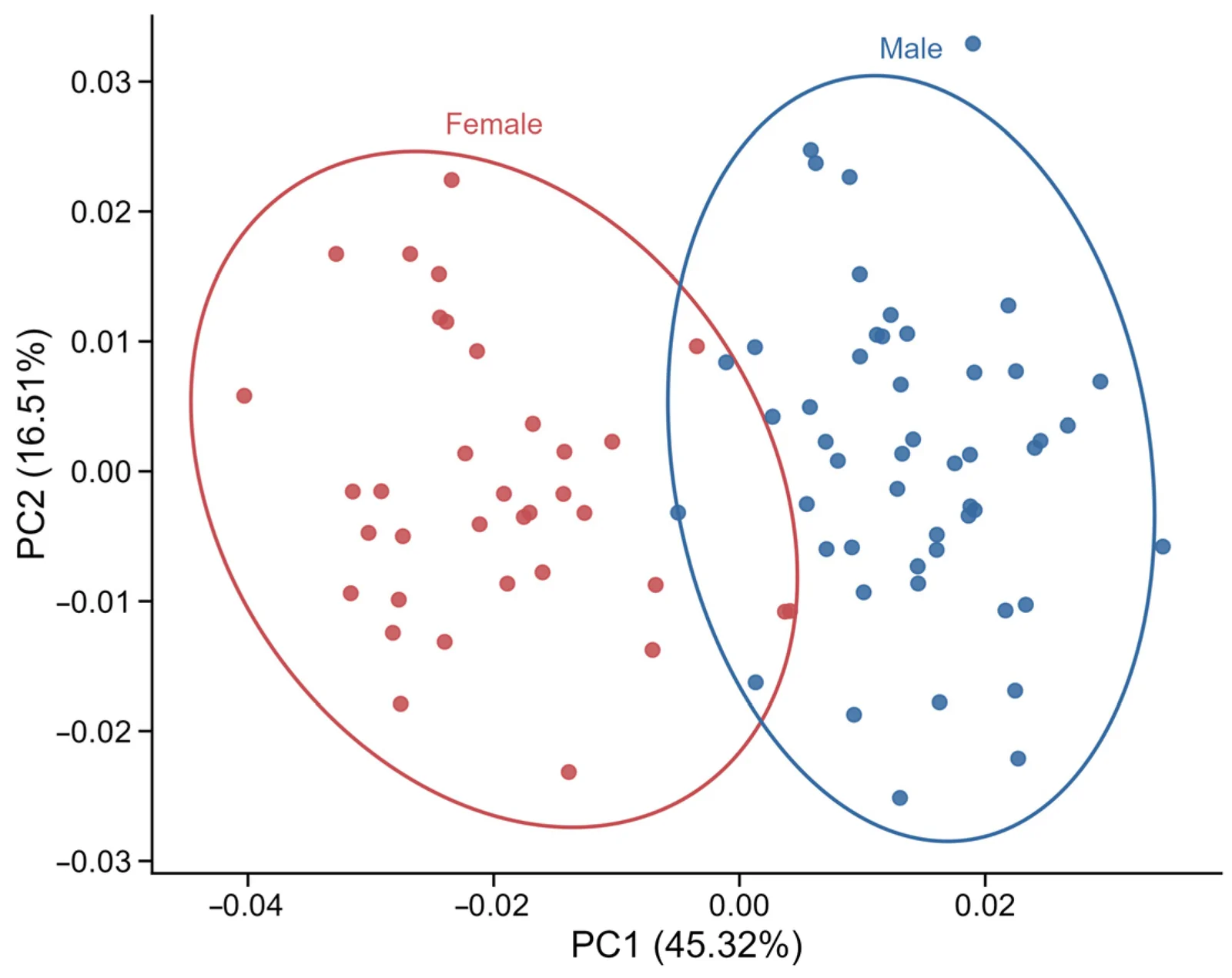
Principal Component Analysis (PCA) of elytral Procrustes coordinates in female and male *Dorcadion parilis*. Ellipses represent 95% group-dispersion regions.

Principal Component Analysis (PCA) of elytral Procrustes coordinates showed that PC1 accounted for 45.32% of total elytral shape variance, whereas PC2 explained 16.51%, together representing 61.83% of the total variation (Figure 5). The first six principal components cumulatively explained 91.45% of elytral shape variance. The PCA scatter plot showed the sex-related structuring of elytral morphospace primarily along PC1, although female and male distributions partially overlapped.

Multivariate analysis of variance (MANOVA), performed on the first six principal components explaining 91.45% of cumulative elytral shape variance, confirmed a highly significant effect of sex on elytral shape (Pillai’s trace = 0.822, F (6, 78) = 60.00, *p* < 0.0001). After Benjamini–Hochberg adjustment across PC1–PC6, the multivariate effect remained attributable primarily to PC1 (F = 295.42, BH-adjusted *p* < 0.0001, η^2^ = 0.781), whereas PC2–PC6 remained non-significant after adjustment. A permutation-based RRPP analysis yielded a consistent result (F = 53.44, R^2^ = 0.392, *p* = 0.0001).

**Figure 6:**
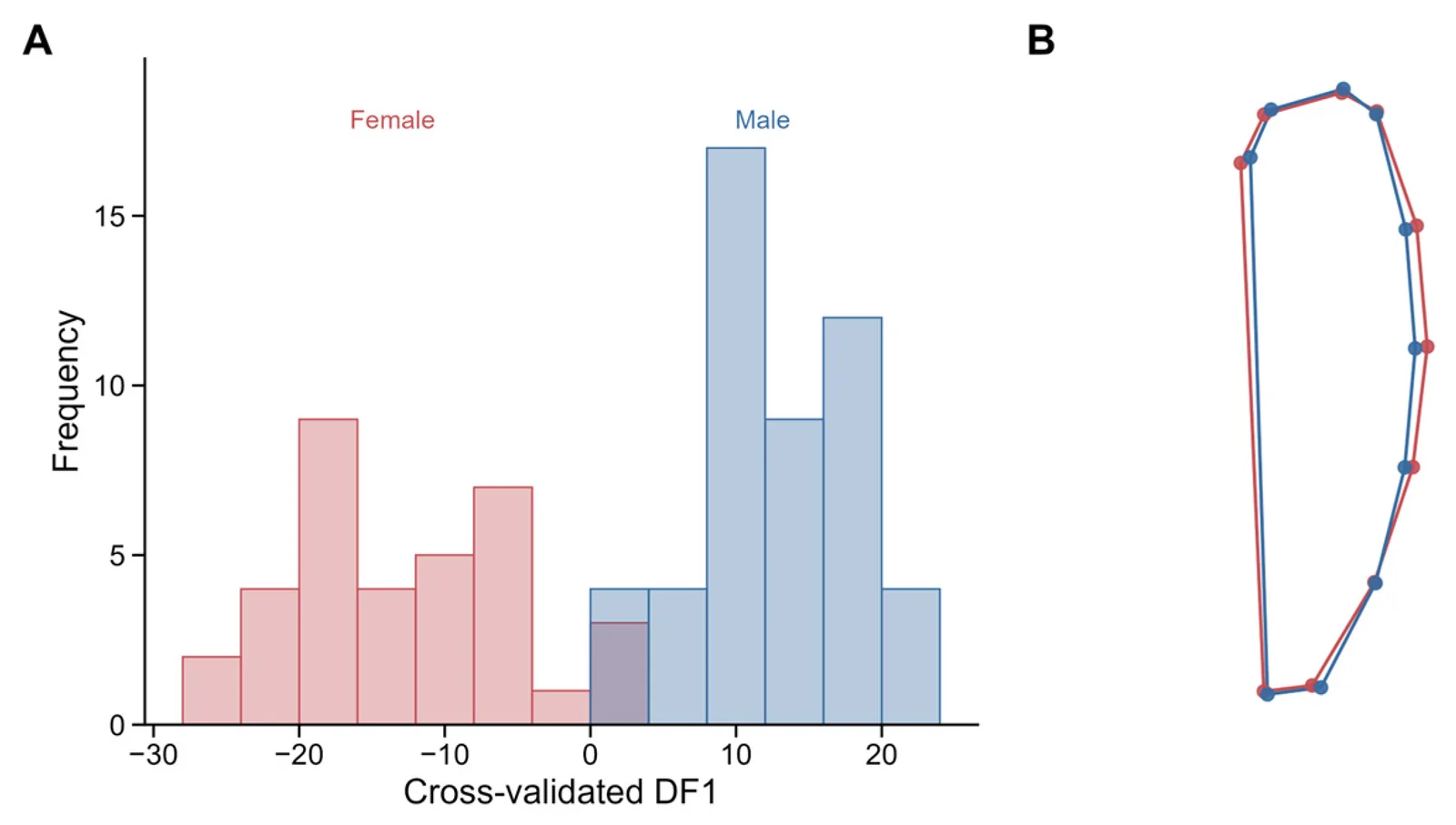
Discriminant Function Analysis (DFA) of elytral shape in female and male *Dorcadion parilis*. (**A**) Distribution of cross-validated discriminant function scores. (**B**) Wireframe comparison of female and male mean Procrustes configurations. Red and blue indicate female and male configurations, respectively.

DFA confirmed significant separation between female and male elytral shapes. Mean shapes differed significantly based on Procrustes distance (0.0342, permutation *p* < 0.001) and Mahalanobis distance (5.1168, permutation *p* < 0.001), with a significant Hotelling’s T^2^ test (T^2^ = 539.03, parametric *p* < 0.001). Cross-validated discriminant function scores showed strong separation with limited overlap (Figure 6A). Original classification accuracy was 100% for both females and males, whereas cross-validation correctly assigned 32/35 females and 50/50 males, corresponding to an overall cross-validated accuracy of 96.5%.

Wireframe comparison of female and male mean Procrustes configurations (Figure 6B) showed subtle but consistent sex-related differences in elytral shape. These differences were most evident along the lateral contour, particularly toward the apical region of the elytra. The female mean configuration appeared slightly broader in this region, whereas the male configuration was relatively more slender.

### 3.3. Pronotal–Elytral Shape Covariation

**Figure 7:**
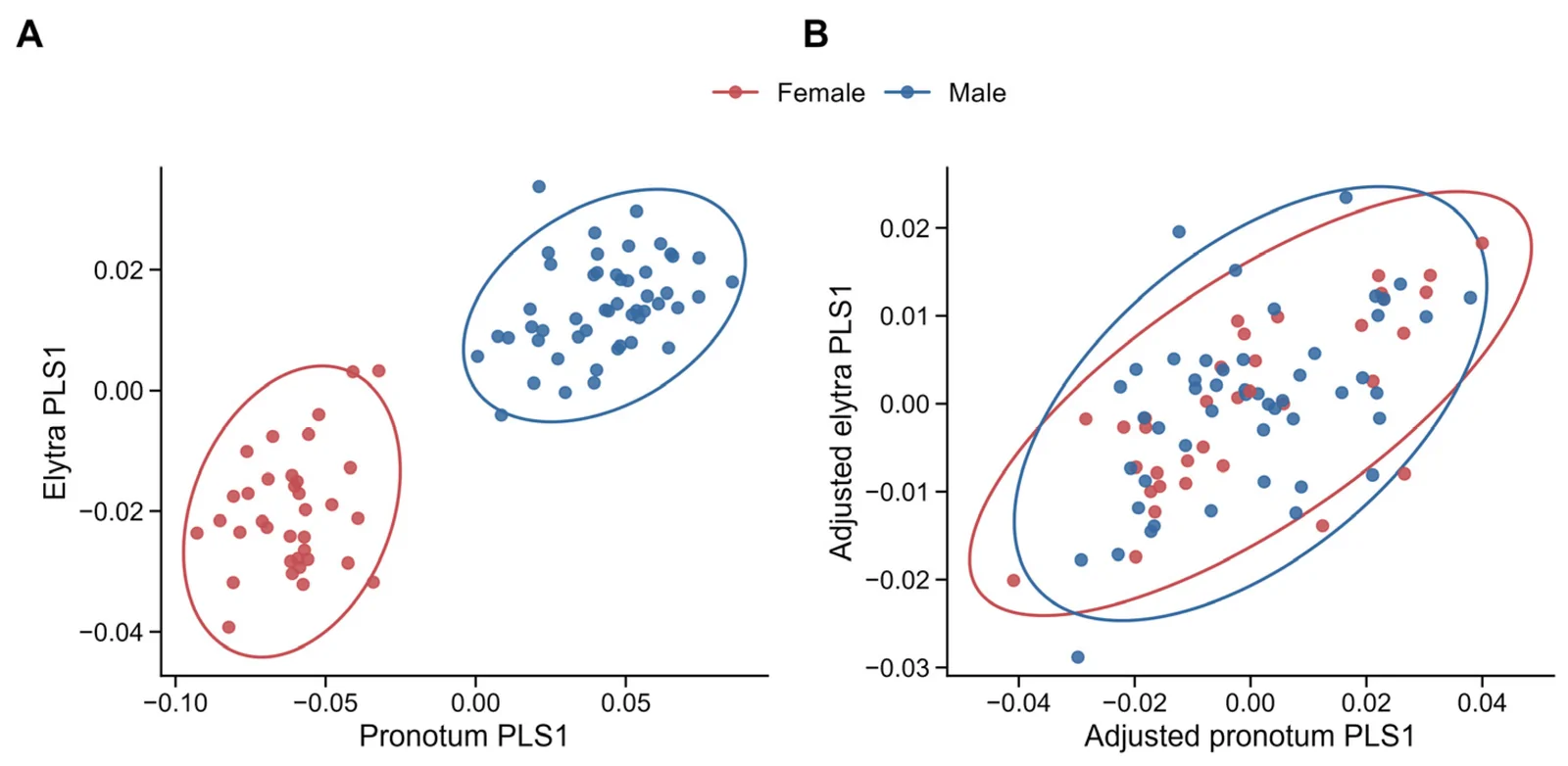
Two-block partial least squares (PLS) analysis of pronotal–elytral shape covariation in female and male *Dorcadion parilis*. (**A**) Unadjusted PLS1 scores. (**B**) PLS1 scores after adjustment for sex and structure-specific log centroid size. Ellipses represent 95% group-dispersion regions.

In the unadjusted two-block PLS analysis, PLS1 accounted for 97.92% of the total between-block squared covariance between the pronotal and elytral shape blocks and showed a strong correlation between block scores (r = 0.8907, permutation *p* = 0.0001; Table 4). The unadjusted PLS scatter plot indicated that this dominant covariation pattern was closely aligned with the sex-related shape differentiation observed in the separate pronotal and elytral analyses (Figure 7A). After sex adjustment, PLS1 accounted for 46.68% of the total between-block squared covariance. The PLS1 singular value remained significant (permutation *p* = 0.0011), and the correlation between block scores was also significant (r = 0.6231, permutation *p* = 0.0001; Table 4). The sex- and size-adjusted PLS produced a comparable result, with PLS1 accounting for 49.52% of the total between-block squared covariance. The PLS1 singular value was significant (permutation *p* = 0.0010), and the block-score correlation remained significant (r = 0.6274, permutation *p* = 0.0002; Table 4; Figure 7B). These results indicate that pronotal–elytral shape covariation was strongly associated with shared sex-related shape variation, while residual shape covariation remained significant after accounting for sex and structure-specific size.

## 4. Discussion

The present study shows that sexual dimorphism in the sampled specimens of *Dorcadion parilis* is expressed through the linear dimensions, relative proportions, and structure-specific shape patterns of the pronotum and elytra. Sexual shape dimorphism was evident in both dorsal structures: pronotal shape showed a more pronounced and spatially broader pattern, whereas elytral shape differentiation was more localized. Although both structures initially showed an apparent allometric signal, centroid size was not supported as an independent source of shape variation after accounting for sex. Pronotal and elytral shapes also covaried, and part of this covariation persisted after accounting for sex and structure-specific centroid size.

External linear measurements and relative proportions have been used to assess sexual differentiation in Cerambycidae [40,41,42] and comparable measurement-based approaches have also been applied in *Dorcadion* taxonomy [25]. These studies indicate that sexual differentiation in longhorn beetles can be expressed through structure-specific dimensions or proportions, rather than only through overall size. In this respect, the present results are consistent with the broader Cerambycidae pattern, but they refine it for *D. parilis* by separating absolute dimensions from Mosimann-adjusted proportional variation. The strongest proportional contrasts involved greater relative pronotal length in males and greater relative elytral width in females, whereas elytral length did not retain a statistically supported proportional difference after size adjustment. This pattern indicates that the linear morphometric signal is organized mainly around male pronotal elongation and female elytral broadening, rather than a generalized enlargement of either sex. The female-biased relative elytral width also provides quantitative support for the original description of *D. parilis*, in which females were characterized by more robust elytra than males [24].

Beyond the proportional differences detected by traditional morphometrics, the GM analyses revealed sex-related shape differentiation in both the pronotum and elytra. This shape-based perspective has become increasingly relevant as the use of GM has expanded in entomology. Landmark-based approaches are now widely used to quantify insect form and to interpret shape variation in relation to taxonomy, intraspecific variation, allometry, and sexual dimorphism [43,44]. In *D. parilis*, pronotal shape showed a clear sex-related pattern, with differences involving the anterior margin and the posterolateral and posterior regions. Males were characterized by a relatively more elongated pronotal configuration, whereas females showed a more compact one. This is consistent with previous work on *Dorcadion* and other Cerambycidae, where pronotal shape has been used to assess sexual dimorphism, population differentiation, or species-level discrimination [26,27,28,29,45]. Elytral shape also differed between sexes, but the deformation pattern was more localized, mainly involving the lateral contour and apical region. Comparable patterns have been reported in Coleoptera, where pronotal and elytral shape do not always show the same degree or pattern of differentiation [13,16,46,47]. Overall, the GM results indicate that sexual shape dimorphism in *D. parilis* involved two main patterns: a broader pronotal pattern and a more localized elytral pattern.

Available evidence from congeners suggests that the pronotal pattern observed in *D. parilis* is unlikely to be unique to this species. In both *D. anatolicum* and *D. micans*, females exhibited wider and shorter pronota, whereas males showed more elongated and sharper pronotal shapes, closely paralleling the pattern found here [27,28]. Although these studies indicate clear pronotal differentiation, its magnitude cannot be compared directly among species because their sample composition and analytical approaches differ. Moreover, the previous study that jointly examined pronotal and elytral shape in *D. micans* included only males and therefore provides no direct comparison of elytral sexual dimorphism [29]. Thus, pronotal dimorphism may represent a recurring pattern within *Dorcadion*, whereas the localized elytral shape differences and female-biased proportional elytral width detected in *D. parilis* remain insufficiently documented across the genus.

The allometric analyses further refine the interpretation of the sex-related shape differentiation detected in the pronotum and elytra. Because allometry can contribute to sexual shape variation in beetles, its role should be assessed before attributing shape differences directly to sex [5,10,48]. In the present study, the initial regressions indicated significant associations between log-transformed centroid size and shape in both structures. However, log-transformed centroid size was not supported as an independent source of shape variation after sex was included in the model. In contrast, sex remained a strong predictor of shape variation, and the log(CS) × sex interaction was not significant for either the pronotum or the elytra. These results indicate that the initial size–shape signal appears to have mainly reflected sex-related structuring in shape space rather than an independent allometric effect. Therefore, sexual shape dimorphism in *D. parilis* should not be interpreted as a secondary consequence of size-related shape change but as a sex-related pattern of shape differentiation that is largely independent of centroid size within the sampled specimens.

Beyond these structure-specific patterns, two-block PLS showed significant covariation between pronotal and elytral shape variation. The unadjusted analysis was dominated by a covariation axis closely aligned with the sex-related separation already observed in the separate pronotum and elytron analyses, indicating that part of the association between the two structures reflected shared sexual dimorphism. However, the association remained significant after accounting for sex and structure-specific log(CS), with similar residual covariation patterns in the sex-adjusted and sex- and size-adjusted analyses. This persistence of residual covariation suggests that the pronotum and elytron share coordinated shape variation beyond the main contrast between females and males. This interpretation is consistent with the use of two-block PLS for evaluating covariation between anatomical structures [20] and with coleopteran geometric morphometric studies showing that pronotal and elytral shape may differ in signal strength while still contributing to broader patterns of interstructural morphological association [15,16,19]. Nevertheless, because the present study is based on external morphology, this pattern should be interpreted as phenotypic covariation rather than as direct evidence of developmental or functional integration. In this respect, the PLS results extend the structure-specific analyses by showing that sexual shape differentiation in *D. parilis* involves both separate pronotal and elytral components and a shared component of dorsal body shape variation.

Taken together, these findings extend previous morphometric work on *Dorcadion* by evaluating sexual dimorphism across multiple analytical levels within a single species-level framework. Earlier studies in the genus have mainly used linear measurements, ratios, or landmark-based shape analyses to address taxonomic differentiation, population-level variation, interspecific differentiation, or sexual dimorphism [25,26,27,28,29]. The present study builds on this work by combining traditional morphometrics, Mosimann-adjusted proportional analyses, geometric morphometrics, sex-controlled allometric evaluation, and two-block PLS analysis of interstructural covariation. This approach is particularly relevant for flightless and taxonomically challenging beetles such as *Dorcadion*, in which external dorsal structures may carry diagnostic information while also expressing sex-related variation. Rather than treating the pronotum and elytra only as isolated taxonomic characters or simple linear dimensions, the present results show that these structures can be examined as structure-specific shape components that also covary with one another.

The present study also has limitations that should guide the interpretation of the results and future research. The analyses were based on external morphology; therefore, the developmental, ecological, functional, behavioral, or genetic processes underlying the observed shape differences could not be directly tested. In addition, reproductive traits, reproductive morphology, and fecundity were not measured, so the relatively broader female elytral configuration should not be interpreted as direct evidence of reproductive performance. The elytral outline was represented by fixed landmarks and equally spaced curve-derived pseudolandmarks, which provided a standardized two-dimensional description of dorsal shape but did not capture three-dimensional curvature or all aspects of surface morphology. Future studies including broader population sampling, ecological variables, reproductive measurements, and complementary three-dimensional or comparative approaches would help clarify whether the observed patterns are stable across populations and whether they have functional or reproductive significance.

## 5. Conclusions

The present study shows that sexual dimorphism in *D. parilis* is expressed as a multidimensional pattern involving linear dimensions, relative proportions, structure-specific sexual shape differentiation, and pronotum–elytra shape covariation. These patterns were not explained simply by centroid size variation; rather, sexual shape differentiation mainly reflected sex-related differences in pronotal and elytral morphology. By integrating traditional morphometrics, geometric morphometrics, sex-controlled allometric assessment, and two-block PLS, this study provides a cautious analytical framework for interpreting sexual dimorphism in *D. parilis*. More broadly, it highlights the value of structure-specific and covariation-based morphometric approaches for studying sexual dimorphism in taxonomically challenging beetle groups.

## Figures and Tables

**Table 1:** Linear morphometric variables in female and male *Dorcadion parilis* based on raw and Mosimann-adjusted measurements.

Trait	Female (Mean ± SD)	Male (Mean ± SD)	F (1, 83)	BH-Adjusted *p*	η^2^
pL	2.17 ± 0.19	2.32 ± 0.17	15.47	<0.001	0.16
pW	3.79 ± 0.32	3.43 ± 0.20	40.46	<0.001	0.33
eL	7.71 ± 0.60	7.09 ± 0.41	32.10	<0.001	0.28
eW	4.97 ± 0.39	4.10 ± 0.22	170.40	<0.001	0.67
pL_adj	0.515 ± 0.019	0.595 ± 0.019	364.76	<0.001	0.81
pW_adj	0.899 ± 0.022	0.879 ± 0.023	15.64	<0.001	0.16
eL_adj	1.830 ± 0.060	1.820 ± 0.042	1.83	0.180	0.02
eW_adj	1.180 ± 0.026	1.050 ± 0.024	533.35	<0.001	0.87

**Table 2:** Sex-controlled Procrustes ANOVA results for pronotal shape in *Dorcadion parilis*.

Effect Tested	df	SS	R^2^	F	*p*
log(CS) after sex	1	0.0014	0.0037	0.73	0.6851
sex after log(CS)	1	0.1721	0.4471	88.44	0.0001
log(CS) × sex	1	0.0010	0.0027	0.52	0.8858

**Table 3:** Sex-controlled Procrustes ANOVA results for elytral shape in *Dorcadion parilis*.

Effect Tested	df	SS	R^2^	F	*p*
log(CS) after sex	1	0.0010	0.0147	1.93	0.0657
sex after log(CS)	1	0.0150	0.2236	29.27	0.0001
log(CS) × sex	1	0.0003	0.0046	0.60	0.7566

**Table 4:** Two-block PLS results for pronotal–elytral shape covariation in *Dorcadion parilis*.

Analysis	PLS1 Singular Value	Squared Covariance (%)	r	*p* (Singular Value)	*p* (Correlation)
Unadjusted PLS	0.000920	97.92	0.8907	0.0001	0.0001
Sex-adjusted PLS	0.000111	46.68	0.6231	0.0011	0.0001
Sex + size-adjusted PLS	0.000112	49.52	0.6274	0.0010	0.0002

Note. *p*-values were obtained from permutation tests. r indicates the correlation between PLS1 block scores.

## Data Availability

The data presented in this study are available from the corresponding author upon reasonable request.
